# The Influence of Isoenzyme Composition and Chemical Modification on Horseradish Peroxidase@ZIF-8 Biocomposite Performance

**DOI:** 10.3390/polym14224834

**Published:** 2022-11-10

**Authors:** Marija D. Stanišić, Nikolina Popović Kokar, Predrag Ristić, Ana Marija Balaž, Miloš Ognjanović, Veljko R. Đokić, Radivoje Prodanović, Tamara R. Todorović

**Affiliations:** 1 University of Belgrade, Faculty of Chemistry, Studentski trg 12-16, 11000 Belgrade, Serbia; 2 Institute of Chemistry, Technology and Metallurgy, National Institute of the Republic of Serbia, University of Belgrade, Njegoševa 6, 11000 Belgrade, Serbia; 3 Institute of Nuclear Sciences “Vinča”, National Institute of the Republic of Serbia, University of Belgrade, Mike Petrovića Alasa 12-14, Vinča, 11000 Belgrade, Serbia; 4 Innovation Center of the Faculty of Technology and Metallurgy, University of Belgrade, Karnegijeva 4, 11000 Belgrade, Serbia

**Keywords:** horseradish peroxidase, isoenzymes, ZIF-8, biomimetic mineralization, biocomposites, biocatalysts, phosphate-acetate buffer influence

## Abstract

Many articles in the literature deal with horseradish peroxidase (HRP) biomineralization, but none pay attention to the isoenzyme composition of commercial HRP or the influence of the carbohydrate component of the protein molecule on the biomineralization process. To study the impact of these factors, we performed periodate oxidation of commercial HRP and a purified HRP-C isoform for biomineralization within ZIF-8. With purified HRP, enzyme@ZIF-8 biocomposites with higher activity were obtained, while periodate oxidation of the carbohydrate component of both commercial HRP and purified HRP-C yields biocomposites with very high activity in acetate buffer that does not degrade the ZIF-8 structure. Using acetate instead of phosphate buffer can prevent the false high activity of HRP@ZIF-8 biocomposites caused by the degradation of ZIF-8 coating. At the same time, purification and especially oxidation of the carbohydrate component of enzymes prior to biomineralization lead to significantly improved activity of the biocomposites.

## 1. Introduction

The enzyme horseradish peroxidase (HRP, EC 1.11.1.7) is a glycoprotein with 18–20% (*w*/*w*) of carbohydrates on the surface [1]. It is the most known plant peroxidase that can be used for various applications such as organic synthesis of specialty chemicals, such as DOPA [2] and phenols [3], removal of various pollutants from wastewaters [3], the manufacture of biosensors [4,5], as well as in various diagnostic kits [6]. In such applications, various immobilization methods are used to increase the stability under operating conditions and enable repeated enzyme use. Therefore, investigations into the influence of the carrier and immobilization method on the stability and activity of immobilized HRP are of special importance for the economic and sustainable application of the immobilized enzyme.

For example, HRP was previously immobilized in hydrogels like alginate macro-beads for azo dye degradation and phenol removal from wastewater [7]. In addition, HRP was immobilized covalently on macroporous methacrylate-based carriers [8]. Although alginate entrapment is a mild method that has no effect on the enzyme activity, to keep the enzyme molecule within the large pores of the polysaccharide gel network, alginate beads with diameters of several millimeters must be used. For this reason, diffusion limitation occurs, which leads to decreased enzyme efficiency. To avoid diffusional constraints, the use of alginate microbeads prepared in an emulsion would be beneficial, but to prevent leakage of an enzyme from the microbeads, additional chemical steps are necessary [9]. One way to decrease HRP leakage from alginate beads is to use chemically modified alginate [10]. Alternatively, biomimetic mineralization can be employed [11,12].

The one-pot in situ biomimetic mineralization method for enzyme encapsulation has been widely studied in the past few years [12]. Most probably due to biocompatible reaction conditions, zeolitic imidazolate framework-8 (ZIF-8) of sodalite (**sod**) topology is frequently used material for enzyme immobilization by encapsulation. Factors that affect biomineralization reactions include concentration and source of Zn(II) ions, pH, temperature, mixing/agitation, etc. [12]. However, the protein surface chemistry may significantly affect biocomposite crystallization since it has been shown that positively charged Zn(II) ions tend to accumulate at proteins’ surfaces [13]. Thus, the electrostatic properties of a protein’s surface, i.e., its pI and zeta potential, can be used to predict whether a protein will induce ZIF-8 growth from an aqueous solution [13]. Indeed, we recently demonstrated that periodate oxidation of carbohydrate components of another industrially relevant glycoprotein-glucose oxidase (GOx), where the negative charge on the enzyme molecule is increased, can facilitate biomimetic mineralization [14]. This process led to the rapid formation of biocomposites with higher thermal stability and activity compared to their native counterparts. However, it is well known that some enzymes exist in different isoforms. For example, for HRP, at least 28 native isoforms have been described so far [15]. HRP isoenzymes are divided into acidic (A), neutral (C), and basic (E) forms [16]. Different isoforms of an enzyme may generate subtle differences in the structure and composition, and consequently also in the activity of the biocomposite [12,13]. Thus, reproduction of the previously published results is often difficult. Current commercial HRP preparations, in the form of lyophilized powders with variable degrees of purity, commonly used in biomimetic mineralization reactions [12] are extracted from *Armoracia rusticana* roots as mixtures of isoenzymes [15]. By high resolution isoelectric focusing, it was shown that three different commercial sources of HRP gave 42 HRP isoforms [17].

Taking into account that previously reported biomineralization studies of HRP did not account for HRP isoform composition [12], in the present study we tested the influence of purification of HRP, as well as oxidation of the carbohydrate component of the protein molecule on the biomineralization process. For the obtained biocomposites, important performance parameters [12,18] pertaining to enzyme immobilization were evaluated. Results showed that purification of HRP and periodate oxidation of the carbohydrate component of protein molecules increased the specific activity of biocomposites. Additionally, periodate oxidation had a greater influence, resulting in biocomposites with significantly increased specific activities and thermostability. Also, it was observed that the presence of unknown protein impurities in the commercial HRP preparation had an important influence on the biomineralization process and performance parameters of biocomposites. Since there is a growing need for biocomposite materials with well-defined properties in the field of enzyme immobilization, this study contributes to the understanding of the biomimetic mineralization process and may provide a blueprint for the modification of protein surface chemistry, and necessary prerequisites in the development of industrially important biocomposite catalysts.

## 2. Materials and Methods

### 2.1. Materials

HRP (lyophilized, powder, ~150 U/mg ABTS units, product No. 77332) and pyrogallol were purchased from Sigma-Aldrich (St. Louis, MO, USA). Carboxymethyl cellulose (CMC) was purchased from Serva Feinbiochemica GmbH and Co (Heidelberg, Germany). Sodium metaperiodate (NaIO_4_) was purchased from VWR Chemicals (Leuven, Belgium). 2-Methylimidazole (HmIM) was purchased from Sigma-Aldrich (St. Louis, MO, USA), while zinc acetate dihydrate (Zn(CH_3_CO_2_)_2_⸱2H_2_O) was purchased from Lachner (Neratovice, Czech Republic). All reagents were of analytical grade and, unless otherwise stated, used as purchased. All solutions were prepared with distilled water.

### 2.2. Instrumentation

Protein concentrations were measured by UV absorbance at 280 nm on a UV-1800 Shimadzu spectrophotometer (Thermo Fischer Scientific, Waltham, MA, USA) using a quartz cuvette with a 1.0 cm path length. For calculations, we used an extinction coefficient for 1% of HRP solutions (10 mg/mL) of 5.8 at 280 nm. It was confirmed that solutions of Zn(II), HmIM, and NaCl did not affect the protein concentration measurements at 280 nm.

The zeta potentials of the samples were measured on a NanoZS90 (Malvern, UK) device at room temperature (25 °C) using disposable zeta cells (DTS 1070). Measurements were performed a minute after the equilibrium time at native pH.

Powder X-ray diffraction (PXRD) data were obtained on a Rigaku Smartlab X-ray diffractometer (*θ*-*θ* geometry; the samples in a horizontal position) in parafocusing Bragg-Brentano geometry using D/teX Ultra 250 strip detector in the 1D standard mode with a CuKα_1,2_ radiation source (*U* = 40 kV and *I* = 30 mA). The PXRD patterns were collected in 5–65° 2*θ* range, with a step of 0.01° min^−1^.

Field-Emission Scanning Electron Microscopy (FESEM) was used for characterization of morphology of biocomposite samples. SEM micrographs were recorded using a FEI Scios 2 DualBeam (Thermo Fisher Scientific, Waltham, MA, USA) instrument operated at an acceleration voltage of 10 kV in a high vacuum. Before the analysis, the samples were coated with a thin layer of Au using a standard sputtering technique.

Elisa Reader (LKB 5060-006) was used for activity measurements. Protein purity was checked by protein electrophoresis (Cleaver Scientific Ltd., Rugby, UK, Cs—300 V). The instrument that was used for isoelectrofocusing is the Pharmacia LKB Multiphor II.

### 2.3. HRP Purification

The purification of commercial HRP preparations was done following a previous protocol [19]. Briefly, the lyophilized HRP was resuspended in 5 mM sodium acetate buffer (pH 4.5) in a final concentration of 5 mg/mL. The sample was dialyzed against 5 mM sodium acetate buffer (pH 4.5) for 24 h at 4 °C. Afterwards, HRP was purified by ion-exchange chromatography using a CMC packed column (5 mL), equilibrated with 5 mM sodium acetate buffer (pH 4.5). After application of dialyzed HRP, the column was first washed with 5 mM sodium acetate buffer to remove unbound proteins. Bound proteins were eluted stepwise using 15 mM, 50 mM, and 100 mM sodium acetate buffer. All fractions were collected and their absorbance was measured at 280 and 405 nm. The purification efficiency was confirmed by SDS-PAGE electrophoresis and isoelectric focusing. The fractions containing active HRP-C isoforms were collected and used in further experiments. The molecular weight of HRP and purified HRP-C was determined by SDS-PAGE electrophoresis using molecular weight markers as standards.

### 2.4. Periodate Oxidation of HRP and HRP-C

Commercial HRP samples (denoted as HRP), at a final concentration of 2 mg/mL, were oxidized with 2.5, 5, and 50 mM NaIO_4_ in 50 mM sodium acetate buffer (pH 5.0) at 4 °C in the dark for 6 h. The excess of NaIO_4_ was removed by adding glycerol (85% *w*/*v*) at a final concentration of 100 mM following incubation for 30 min. Afterwards, periodate oxidized HRP samples were dialyzed against distilled water at 4 °C for 24 h. Native electrophoresis was performed according to standard procedures. Protein bands were visualized by Coomassie Brilliant Blue staining. HRP-C isoforms (denoted as HRP-C) were oxidized using 2.5 mM NaIO_4_ as described above. For further biomineralization experiments, only samples oxidized with 2.5 mM NaIO_4_ were used (ox-HRP and ox-HRP-C).

### 2.5. Biomimetic Mineralization Experiments

The stock solution of the enzymes (HRP, ox-HRP, HRP-C, and ox-HRP-C), at a final concentration of 0.5 mg/mL, was mixed with Zn(CH_3_CO_2_)_2_⸱2H_2_O (in the final concentration of 0.02 M) for 5 min and then HmIM was added to the solutions in the final concentration of 1.0 M. In order to determine the optimal reaction time, each mixture was stirred on a magnetic stirrer for several periods (30 min, 1 h, 2 h, 4 h, 6 h, and 24 h) and then left at room temperature for 1 h. The precipitates were centrifuged at 6000 rpm for 10 min. The supernatants were collected for protein concentration and activity measurements. The precipitates were collected and washed with distilled water three times.

All precipitates were incubated in 1 M NaCl for 30 min to remove proteins adsorbed on the surface of biocomposites. After incubation in NaCl, all precipitates were collected by centrifugation at 6000 rpm and washed with distilled water three times. The supernatants were collected for protein concentration and activity measurements.

For activity measurements (*vide infra*), the samples obtained during 30 min reaction times were used.

### 2.6. Activity Measurements

The activities of soluble enzymes and biocomposite suspensions were measured using pyrogallol as a substrate. Biocomposites were re-suspended in distilled water at a final concentration of 20 mg/mL. The reaction mixture for measuring the enzyme activity contained 0.1 *w/v* H_2_O_2_ and 0.2 *w/v* pyrogallol (in a 0.1 M sodium acetate buffer, pH 5.5). The activity was measured by monitoring a change in absorbance at 405 nm using an extinction coefficient of 12 mM^−1^ cm^−1^ for oxidized pyrogallol. One unit (1 U) of enzyme activity was defined as the amount of enzyme that converts 1 mg of pyrogallol in 20 s. The same procedure was used for the activity measurements of soluble enzyme samples in potassium phosphate buffer (pH 6.0). The specific activity is expressed as U/mg_protein_ or U/g_biocomposite_.

### 2.7. Thermal Stability

Thermostability of soluble enzymes and biocomposites (washed with distilled water and 1 M NaCl) is expressed as residual activity (in %), which was calculated as the ratio of the specific activities of the samples before and after incubation in water at 60 °C for 1 h.

## 3. Results

### 3.1. Purification, Oxidation, and Zeta Potential of HRP Samples

**Figure 1:**
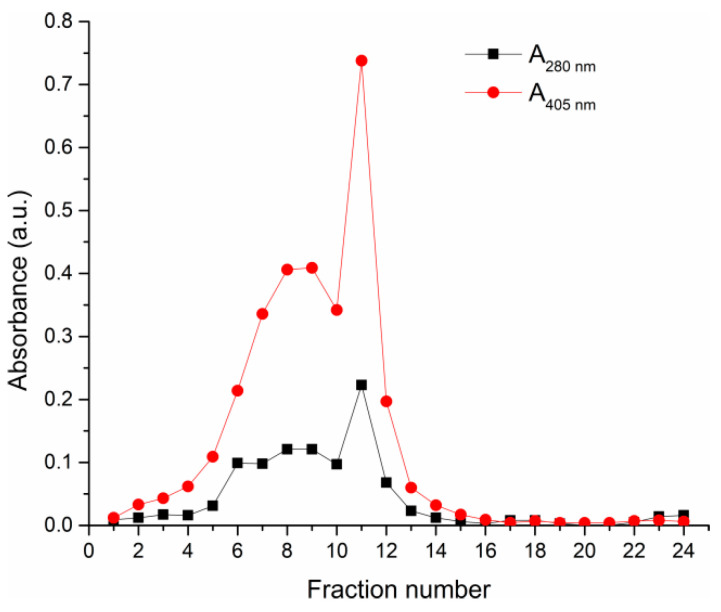
Elution profile of commercial HRP preparation from the CMC chromatographic column. Elution system: equilibration buffer—5 mM sodium acetate buffer (pH 4.6), elution buffers—15 mM and 50 mM sodium acetate buffers (pH 4.6) and 100 mM sodium acetate buffer (pH 5.5).

In order to test the influence of HRP preparation purity on the biomineralization process and activity of biocomposites, HRP-C isoforms were isolated, based on previous protocols, using CMC ion-exchange chromatography [19]. HRP-C isoforms were eluted using increasing concentrations of sodium acetate buffer with different pHs (Figure 1).

**Figure 2:**
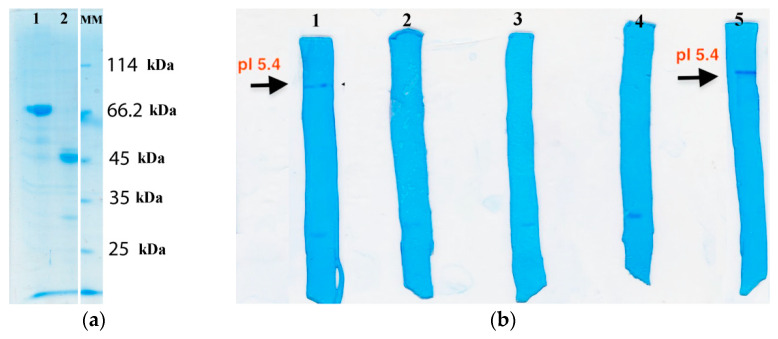
(**a**) SDS-PAGE electrophoresis of: 1—commercial HRP before purification; 2—HRP-C isoform; MM—Molecular weight markers. (**b**) Isoelectric focusing and pI values of HRP isoforms: 1—commercial HRP before purification; 2–4—basic isoforms; 5—HRP-C isoform.

Fractions containing the highest enzyme activity were pooled and HRP-C purity was analyzed by SDS-PAGE electrophoresis (Figure 2a). It could be seen, by comparing the position of protein bands in the purified HRP sample (lane 2, Figure 2a) with molecular weight markers (lane MM, Figure 2a), that the purification procedure removed impurities with a molecular weight of ~70kDa from the commercial HRP sample (lane 1, Figure 2a), and that the obtained HRP-C had an expected molecular weight ~45 kDa. In the commercial HRP sample, there is also a visible protein band at 45 kDa (lane 1, Figure 2a) confirming the molecular weight of HRP at 45 kDa, as expected. The pI value of 5.4 was confirmed by isoelectric focusing (Figure 1b). These results confirmed that HRP-C was successfully purified.

**Figure A1:**
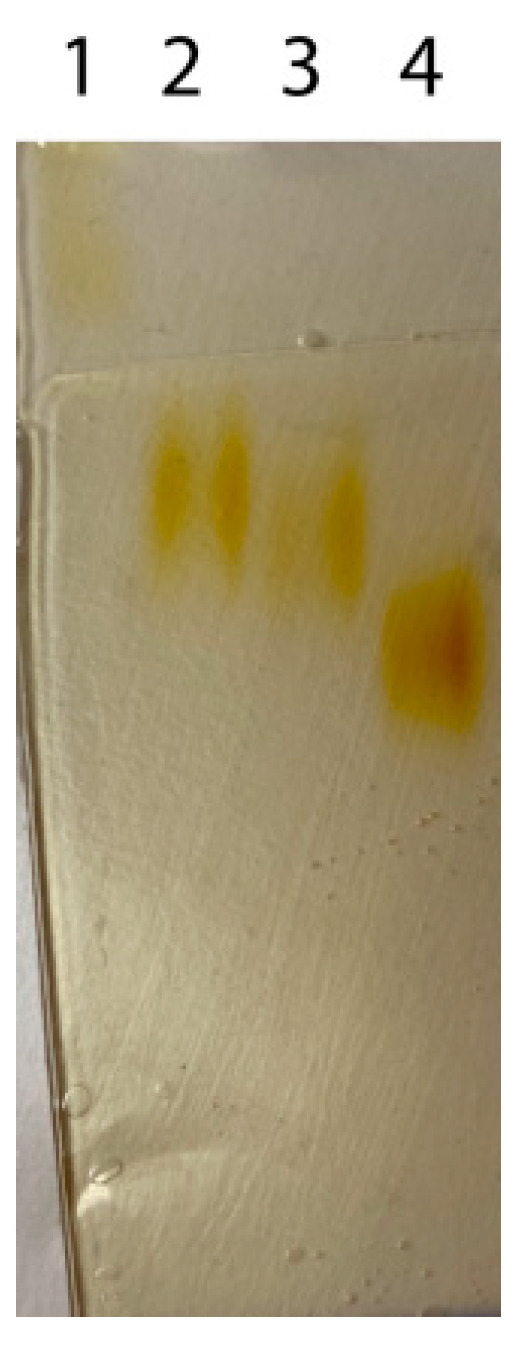
Native electrophoresis of commercial HRP and activity bands in gels after incubation in 5% pyrogallol: 1—commercial HRP, 2–ox-HRP (2.5 mM NaIO_4_), 3–ox-HRP (5.0 mM NaIO_4_), 4–ox-HRP (50.0 mM NaIO_4_).

Since HRP is a glycoprotein that contains 20% (*w*/*w*) carbohydrates on the surface [1], its polysaccharide chains are often used in chemical modification/crosslinking reactions. It is well known that periodate oxidation of its sugar residues generates reactive aldehyde groups that can be used for conjugation to amine-containing molecules [20]. However, we used periodate oxidation in order to increase the negative charge on the protein surface and to facilitate Zn^2+^ ion binding in a one-pot biomineralization process. In further periodate oxidation experiments, both non-purified commercial HRP and purified HRP-C were used. Oxidation of HRP was done using NaIO_4_ at a range of concentrations (2.5 mM to 50 mM) and the change in charge was confirmed by native electrophoresis (Figure A1, Appendix A). The recent study points to the fast decomposition of **sod**-ZIF-8 in phosphate buffer saline media [21], which could be of relevance during precise quantification of the activity of biocomposites containing ZIF-8. Thus, we have compared the specific activities of HRP, HRP-C, and oxidized forms of these enzymes in two buffer systems−potassium phosphate buffer and sodium acetate buffer (Table 1). The specific activities of all enzyme forms were slightly different in the two buffer systems used. The specific activity increased from 13.75 U/mg for non-purified HRP to 64.42 U/mg for purified HRP-C, measured in sodium acetate buffer (pH 5.5). To prevent possible degradation of ZIF-8 coating, although the specific activity of all enzymes was slightly higher in the phosphate buffer, all further activity measurements were done in acetate buffer (pH 5.5).

Since the surface electrostatic potential of enzymes/proteins can be correlated to the zeta potential [13], we next measured the zeta potential of non-oxidized and oxidized HRP and HRP-C samples (Table 2). Moreover, we monitored the impact of the addition of Zn(II) ions/HmIM on the zeta potential of all enzymes (Table 2). Based on the zeta potential data, we chose a 2.5 mM NaIO_4_ concentration as the most appropriate for oxidation of both HRP and HRP-C, since the most negative charge on the surface of oxidized enzymes was obtained with this concentration of the oxidant. The addition of HmIM to all non-oxidized enzyme solutions decreased the zeta potentials slightly, while for oxidized forms, zeta potentials increased by about 50%, making the protein surface more positive. Thus, further biomimetic mineralization experiments were done by mixing the enzyme solutions with solutions containing Zn(II) ions first, and afterwards HmIM was introduced. This way of mixing of the reactants enables the effective accumulation of Zn(II) ions on the enzyme surface that engenders biomimetic mineralization.

### 3.2. Biomimetic Mineralization of HRP Samples

**Figure 3:**
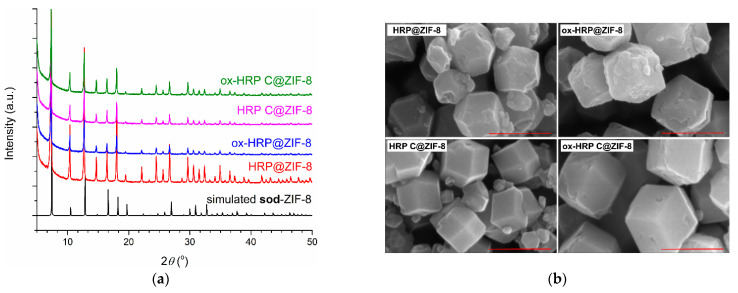
(**a**) Simulated PXRD pattern of the **sod**-ZIF-8 and experimental PXRD patterns of the enzyme@ZIF-8 samples obtained during 30 min, washed with distilled water and air dried; (**b**) SEM images of four types of biocomposites (scale bar 500 nm).

**Figure A2:**
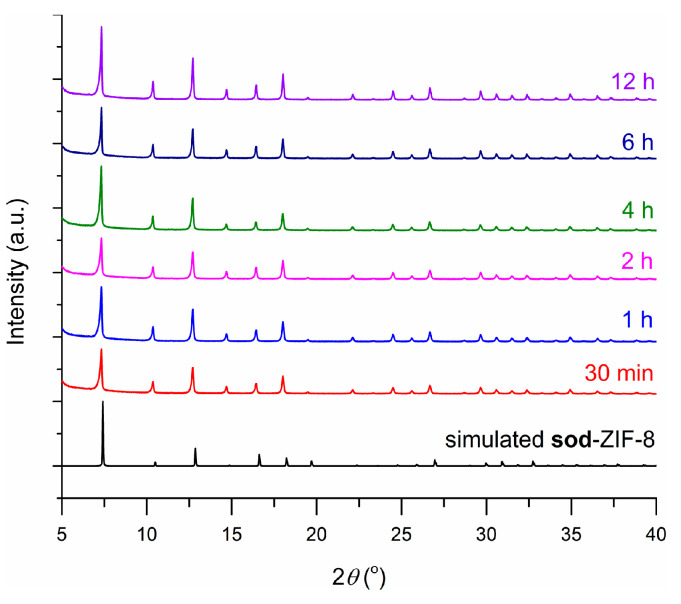
Simulated PXRD pattern of the **sod**-ZIF-8 and experimental PXRD patterns of HRP@ZIF-8 samples obtained during different reaction times.

**Figure A3:**
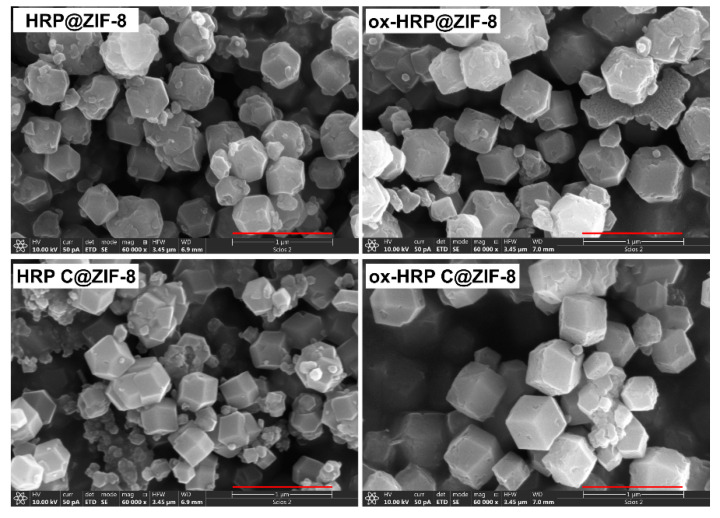
SEM images of bulk samples of four types of biocomposites (scale bar = 1 μm).

For the optimization of reaction time, biomineralization experiments (room temperature in distilled water) with HRP were performed with mixing times of 30 min, 1 h, 2 h, 4 h, 6 h, and 12 h, followed by agitation for 1 h. In all these experiments, the final enzyme concentration was 0.5 mg/mL, while the final concentrations of Zn(II) ions and HmIM were 0.02 M and 1.0 M, respectively. These precursor concentrations/mole ratios are prerequisites for the synthesis of phase pure biocomposites with preferred **sod**-ZIF-8 topology [22]. The precipitates were washed with distilled water, air dried, and further examined by PXRD. Obtained PXRD patterns were compared to the simulated ones for **sod**-ZIF-8 (Figure A2, Appendix A). It was confirmed that in all reactions biocomposites with **sod** ZIF-8 topology were obtained. For further experiments, a reaction time of 30 min was chosen. Figure 3a shows PXRD patterns of the precipitates obtained using HRP, ox-HRP, HRP-C, and ox-HRP-C, while SEM images (Figure 3b) confirmed that standard rhombic dodecahedral microcrystalline biocomposites, exhibiting average crystallite diameters of 400–550 nm, were obtained. From SEM images of bulk samples (Figure A3, Appendix A), the following size distribution of biocomposites was measured: 392 ± 37 nm (HRP@ZIF-8), 399 ± 41 nm (HRP-C@ZIF-8), 546 ± 54 nm (ox-HRP@ZIF-8), and 543 ± 71 nm (ox-HRP-C@ZIF-8).

### 3.3. Performance Parameters of Biocomposites

The performance parameters for the biocomposites were determined and the results are shown in Table 3. These are the following: immobilization yield (*Y*) for activity (*A*) and protein (*P*) which is distributed between the liquid and the solid phase; activity balance (*Y_A_*; the ratio of total immobilized enzyme activity, the surface attached plus encapsulated one, and activity of the enzyme used for the biomineralization); protein balance (*Y_P_*; the ratio of the total amount of immobilized protein and the amount of protein used for the biomineralization); protein loading (*P*_loading_; mass of immobilized protein in mg per one g of the carrier); specific activity (U/g_biocomposite_); specific activity of the bound enzyme (U/mg_enzyme bound_); effectiveness factor (*η*; the ratio of the specific activity of the bound enzyme and specific activity of the free soluble enzyme) [12,18]. The parameters were determined for the biocomposites washed with water alone and for the ones washed with water and 1 M of NaCl.

HRP@ZIF-8 had a very low specific activity of only 0.61 U/g of the biocomposite when measured in acetate buffer, and a very low activity balance, even though protein loading was ~58 mg/g. This was also accompanied with a very low specific activity of bound enzyme (<0.01 U/mg). When a carbohydrate component of the commercial HRP was oxidized using 2.5 mM NaIO_4_ and then biomineralized (ox-HRP@ZIF-8), a drastic increase in specific activity of up to ~125 U/g was observed. The protein balance (0.77) and loading (90.4 mg/g_carrier_) were also higher compared to the non-oxidized HRP sample (HRP@ZIF-8), as well as an activity balance of 0.76 and specific activity of bound enzyme of ~1.4 U/mg.

Biocomposite with HRP-C (HRP-C@ZIF-8), with a higher specific activity of 3.48 U/g compared to the biocomposite with non-purified commercial HRP (HRP@ZIF-8), was obtained. This is in accordance with a higher activity balance of 0.09 and specific activity of the bound enzyme of 0.12 U/mg compared to HRP@ZIF-8. Oxidation of the carbohydrate moiety of the purified HRP-C prior to biomineralization again resulted in biocomposite (ox-HRP-C@ZIF-8) with a drastically increased specific activity of ~50 U/g, accompanied by higher activity balance (0.97) and specific activity of bound enzyme (0.66 U/g), compared to the biocomposite with non-oxidized HRP-C (HRP-C@ZIF-8).

Washing the biocomposites with a 1 M NaCl solution decreased the specific activity (U/g_biocomposite_) of all of the samples. This is likely due to the removal of the surface-bound protein molecules. We could notice that this effect had the least influence in the case of ox-HRP-C@ZIF-8, where the specific activity of the washed biocomposite was only slightly decreased to 47.2 U/g.

During storage of the biocomposite samples at 4 °C for two weeks, there was no drop in activity.

### 3.4. Thermostability Studies

To determine the influence of purification and carbohydrate oxidation on the thermal stability of the enzymes, thermostability studies for both soluble enzymes and biocomposites after a 1 h incubation at 60 °C were performed. The results are expressed as residual activity (%) defined as the ratio of the specific activity after and before incubation at 60 °C for 1 h. The residual activity of soluble HRP, HRP-C, ox-HRP, and ox-HRP-C was 35, 48, 35, and 42%, respectively. Thus, all enzymes are significantly deactivated upon heating. As demonstrated in the results given in Table 4, the thermostability of purified and oxidized HRP-C was significantly increased after biomineralization. The biocomposite ox-HRP-C@ZIF-8 did not show a drop in activity even after washing with NaCl followed by heating for 1 h at 60 °C. This was in accordance with our previous findings that periodate oxidation of the carbohydrate moiety of enzyme molecules, when performed before biomineralization, leads to biocomposites with much higher activity and thermostability [14].

**Figure A4:**
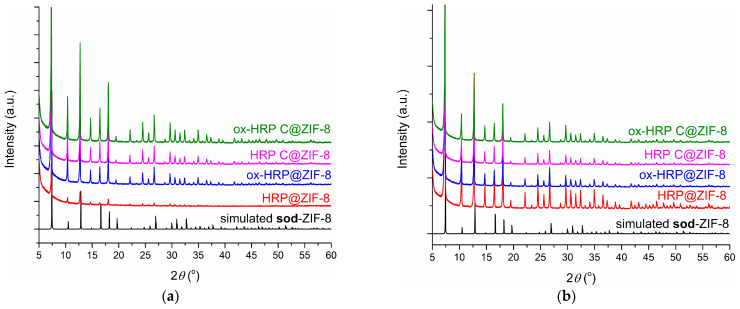
(**a**) Simulated PXRD pattern of the **sod**-ZIF-8 and experimental PXRD patterns of the biocomposite samples washed only with water after incubation at 60 °C for 1 h; (**b**) Simulated PXRD pattern of the **sod**-ZIF-8 and experimental PXRD patterns of the biocomposite samples washed with water and a 1 M NaCl solution after incubation at 60 °C for 1 h.

During the thermal treatment, all biocomposites remained intact in terms of ZIF-8 coating topology as determined by PXRD (Figure A4, Appendix A).

## 4. Discussion

The specific activity of commercial HRP immobilized within ZIF-8, when measured in acetate buffer, was much lower compared to data from previous literature for HRP@ZIF-8 biocomposites [12], where the enzyme activity was measured in phosphate buffer. The reason for this could be the fact that phosphate can disintegrate the ZIF-8 structure [21] and could release some of the encapsulated HRP into the solution and/or make the ZIF-8 coating more porous, thus leading to falsely higher values of specific activity of the biocomposites.

Due to diffusional limitations, biocomposites usually have lower specific activities compared to free soluble enzymes [12]. This was observed in our case for purified HRP-C. In the case of commercial HRP, due to the presence of large amounts of protein impurities (see lane 1, Figure 2a), almost none of the HRP was biomineralized (neither on the surface nor inside ZIF-8).

Oxidizing the carbohydrate component of commercial HRP with 2.5 mM NaIO_4_, followed by biomineralization, drastically increased the specific activity of the biocomposite, similar to the results that we obtained with glucose oxidase [14]. The results are in accordance with a higher protein balance and loading compared to the biocomposite with non-oxidized HRP. In addition, the activity balance and specific activity of the bound enzyme were much higher. The results showed that our biomineralization method, where the first step is oxidation of the carbohydrate component of the enzyme molecule, yields biocomposites with very high activity, not previously reported in the literature for HRP, when phosphate degradation of the ZIF-8 coating was prevented.

The HRP-C biocomposite showed higher specific activity compared to the biocomposite with commercial HRP. Correspondingly, higher activity balance and specific activity of bound enzyme were also obtained for the HRP-C@ZIF-8 compared to the non-purified commercial HRP biocomposite. These results imply that purity and isoenzyme composition of commercial HRP, with tens of different isoforms [15,17], can significantly influence the biomineralization experiments. In previous similar biomineralization studies with commercial HRP preparations [12,23,24,25,26], the purity and isoform composition were not tested or taken into account.

The oxidation of the carbohydrate moiety of the purified HRP-C prior to biomineralization yields a biocomposite with drastically increased specific activity, accompanied by a higher activity balance and specific activity of the bound enzyme, compared to the non-oxidized HRP-C biocomposite.

As expected, washing the biocomposites with NaCl decreases the specific activity of all tested biocomposites, likely due to the removal of the surface-bound enzyme molecules. The removal of surface bound protein molecules was less pronounced in the case of ox-HRP@ZIF-8 compared to the ox-HRP-C@ZIF-8 biocomposite (see *P*_loading_ values, Table 3). However, NaCl washing removed much greater amounts of enzyme activity in the case of ox-HRP@ZIF-8 (around 50%; see specific activity values, Table 3). In the case of ox-HRP-C@ZIF-8, washing with NaCl removed very low amounts of the enzyme activity (around 4%). It seems that, contrary to the ox-HRP, most of the ox-HRP-C enzyme molecules were encapsulated within the biocomposite. This was also corroborated by the results of the thermostability studies, where ox-HRP-C@ZIF-8 (washed with water and NaCl) preserved activity after incubation at 60 °C for 1 h. This is in line with previous findings where periodate oxidized invertase was immobilized by adsorption on sepiolite [27]. The oxidized adsorbed invertase was more tightly bound to the inorganic carrier, thus more resistant to washing out by concentrated salt solution. Also, it had a higher half-life at 60 °C than adsorbed native enzyme. It could be concluded that protein impurities present in the commercial HRP preparations can influence the biomineralization process and efficiency of enzyme encapsulation.

Taking into account all of our results, a distribution of enzymes can be assumed. Almost all of the commercial HRP remained in the supernatant after biomineralization under applied conditions. Similarly, ~80% of the initial amount of HRP-C remained in the supernatant. The bound HRP-C was predominantly located on the surface of ZIF-8. Periodate oxidation of commercial HRP increases the amount of bound protein by ~30%, but enzyme molecules are mainly located on the surface of ZIF-8. Remarkably, ~97% of the initial amount of ox-HRP-C was bound to the ZIF-8. Washing with NaCl removed half of the bound protein. Thermostability studies of the ox-HRP-C@ZIF-8 biocomposite washed only with water and additionally with NaCl imply that the active enzyme molecules were located mostly inside ZIF-8.

To sum up, during biomineralization of HRP or other proteins/enzymes, the purity and the presence of other proteins should be taken into account. Periodate oxidation of purified HRP prior to biomineralization yields biocomposites with higher specific activities and thermostability. This is in accordance with our previous findings with GOx [14]. Also, one should pay attention to the buffer composition used for determination of performance parameters of enzyme@ZIF-8 biocomposites in order to avoid artefacts.

## Figures and Tables

**Table 1:** Specific activities (U/mg) of commercial HRP, its oxidized form, purified HRP-C, and oxidized HRP-C in phosphate and acetate buffers.

Sample	Specific Activity (U/mg) Phosphate Buffer (pH 6.0)	Specific Activity (U/mg) Acetate Buffer (pH 5.5)
HRP	18.83 ± 0.80	13.75 ± 0.58
ox-HRP (2.5 mM NaIO_4_)	2.89 ± 0.21	2.17 ± 0.17
HRP-C	81.80 ± 4.42	64.42 ± 3.30
ox-HRP-C (2.5 mM NaIO_4_)	49.52 ± 3.10	41.25 ± 2.60

**Table 2:** Experimental zeta potentials of the tested enzyme solutions. Conditions: 0.35 mg/mL of enzymes in distilled water, 0.02 M for Zn(II) solutions or 1.00 M for HmIM solutions.

Sample	Zeta Potential (mV)
HRP	−26.7 ± 2.6
HRP in Zn(II) solution	+2.82 ± 0.75
HRP in HmIM solution	−28.1 ± 3.6
ox-HRP (2.5 mM NaIO_4_)	−46.7 ± 4.6
ox-HRP (5.0 mM NaIO_4_)	−40.2 ± 5.9
ox-HRP (50.0 mM NaIO_4_)	−34.4 ± 3.8
ox-HRP in Zn(II) solution	+2.72 ± 0.84
ox-HRP in HmIM solution	−24.4 ± 3.2
HRP-C	−27.3 ± 3.4
HRP-C in Zn(II) solution	−2.84 ± 1.15
HRP-C in HmIM solution	−29.1 ± 2.6
ox-HRP-C	−47.5 ± 5.2
ox-HRP-C in Zn(II) solution	+2.84 ± 1.24
ox-HRP-C in HmIM solution	−23.8 ± 2.7

**Table 3:** Key immobilization performance parameters for four types of biocomposites, washed with water alone or washed with water and a 1 M NaCl solution (data in parentheses) *.

Parameter	HRP@ZIF-8	ox-HRP@ZIF-8	HRP-C@ZIF-8	ox-HRP-C@ZIF-8
*Y*_activity balance_	<0.01	0.76 ± 0.05	0.09 ± 0.05	0.97 ± 0.01
	(<0.01)	(0.75 ± 0.05)	(0.09 ± 0.04)	(0.97 ± 0.01)
*Y*_protein balance_	0.49 ± 0.05	0.77 ± 0.06	0.23 ± 0.01	0.97 ± 0.02
	(0.27 ± 0.07)	(0.71 ± 0.01)	(0.11 ± 0.08)	(0.54 ± 0.21)
*P*_loading_ (mg/g_carrier_)	57.52 ± 3.37	90.39 ± 6.01	27.01 ± 9.51	76.10 ± 5.59
	(38.01 ± 7.70)	(83.11 ± 1.49)	(10.61 ± 6.63)	(43.28 ± 18.19)
Specific activity (U/g_biocomposite_)	0.61 ± 0.06	125.37 ± 3.06	3.48 ± 2.55	49.92 ± 13.95
	(0.40 ± 0.05)	(62.80 ± 5.82)	(2.20 ± 0.33)	(47.16 ± 15.41)
Specific activity (U/mg_enzyme bound_)	<0.01	1.39 ± 0.08	0.12 ± 0.04	0.66 ± 0.21
	(<0.01)	(0.76 ± 0.04)	(0.26 ± 0.16)	(1.43 ± 1.21)
*η* × 100 (%)	<0.07	64.06 ± 4.05	0.19 ± 0.05	1.60 ± 0.52
	(<0.07)	(35.02 ± 3.15)	(0.40 ± 0.07)	(3.47 ± 0.32)

* Data given in parentheses refer to biocomposites washed first with water and then with a 1 M NaCl solution.

**Table 4:** Thermal stability of four types of biocomposites washed with water, and additionally with a 1 M NaCl solution, expressed as residual activity (%; defined as a ratio of specific activity after and before incubation at 60 °C for 1 h). Residual activity of soluble HRP, HRP-C, ox-HRP, and ox-HRP-C, incubated at 60 °C for 1 h, was 35, 48, 35, and 42%, respectively.

Parameter	HRP@ZIF-8	ox-HRP@ZIF-8	HRP-C@ZIF-8	ox-HRP-C@ZIF-8
Residual activity (%) of samples washed with water	64.20 ± 20.34	57.61 ± 28.84	35.18 ± 12.52	102.32 ± 22.07
Residual activity (%) of samples washed with water and 1 M NaCl	55.78 ± 7.94	41.71 ± 6.86	20.81 ± 2.60	119.50 ± 3.93

## Data Availability

The data presented in this study are available upon request from the corresponding author.
